# Supplementary material for: SyNDI: synchronous network data integration framework
Source: BMC Bioinformatics. 2018 Nov 6;19:403. doi: 10.1186/s12859-018-2426-5 (PMC6219086; doi:10.1186/s12859-018-2426-5)
Supplement: Supplementary file 5 — Files of the example presented in Additional file 4. This additional file is a zip package that contains all files used in Additional file 4. Detailed descriptions of each of these files are presented in a README file included in this zip package. (GZ 30411 kb) [file 12859_2018_2426_MOESM5_ESM.gz › SyNDITools/Meme2Fimo/meme_4.9.0_4.tar.gz!/meme_4.9.0/doc/cite.html]

Citing MEME and MAST


|  |  |
| --- | --- |
|  | MEME Suite The MEME Suite web application requires the use of JavaScript   Javascript doesn't seem to be available on your browser.  To cite the full MEME Suite   - Timothy L. Bailey, Mikael Bodén, Fabian A. Buske, Martin Frith, Charles E. Grant, Luca Clementi,   Jingyuan Ren, Wilfred W. Li, William S. Noble,   "MEME SUITE: tools for motif discovery and searching",   *Nucleic Acids Research*, 37:W202-W208, 2009.   [full text]   To cite individual tools   - **AMA** Fabian A. Buske, Mikael Bóden, Denis C. Bauer and Timothy L. Bailey,   "Assigning roles to DNA regulatory motifs using comparative genomics",   *Bioinformatics*, 26(7):860-866, 2010.   [full text] - **AME** Robert C. McLeay, Timothy L. Bailey,   "Motif Enrichment Analysis: a unified framework and an   evaluation on ChIP data",   *BMC Bioinformatics*,   11:165, 2010.   [full text] - **CentriMo** Timothy L. Bailey and Philip Machanick,   "Inferring direct DNA binding from ChIP-seq",   *Nucleic Acids Research*, **40**:e128, 2012.   [Abstract and Full Text] - **DREME** Timothy L. Bailey,   "DREME: Motif discovery in transcription factor ChIP-seq data",   *Bioinformatics*, 27(12):1653-1659, 2011.   [full text] - **FIMO** Charles E. Grant, Timothy L. Bailey, and William Stafford Noble,   "FIMO: Scanning for occurrences of a given motif",   *Bioinformatics* 27(7):1017–1018, 2011.   [full text] - **GLAM2 and GLAM2SCAN** Martin C. Frith, Neil F. W. Saunders, Bostjan Kobe, Timothy L. Bailey,   "Discovering sequence motifs with arbitrary   insertions and deletions",   *PLoS Computational Biology*, **4**(5):e1000071, 2008.   [full text] - **GOMO** Fabian A. Buske, Mikael Boden, Denis C. Bauer and Timothy L. Bailey,   "Assigning roles to DNA regulatory motifs using comparative genomics",   *Bioinformatics*, 26(7), 860-866, 2010.   [full text] - **MAST** Timothy L. Bailey and Michael Gribskov,   "Combining evidence using p-values: application to sequence homology searches",   *Bioinformatics*, 14(1):48-54, 1998.   [pdf] - **MEME** Timothy L. Bailey and Charles Elkan,   "Fitting a mixture model by expectation maximization to   discover motifs in biopolymers",   *Proceedings of the Second International Conference on Intelligent Systems   for Molecular Biology*, pp. 28-36, AAAI Press, Menlo Park, California, 1994.   [postscript]   [pdf] - **MEME-ChIP** Philip Machanick and Timothy L. Bailey,   "MEME-ChIP: motif analysis of large DNA datasets",   *Bioinformatics* 27(12):1696-1697, 2011.   [full text] - **PSPs** Timothy L. Bailey, Mikael Bodén, Tom Whitington, and Philip Machanick,   "The value of position-speciﬁc priors in motif   discovery using MEME",   *BMC Bioinformatics*, 11(1):179, 2010.   [full text] - **MCAST** Timothy Bailey and William Stafford Noble,   "Searching for statistically significant regulatory modules",   *Bioinformatics (Proceedings of the European Conference on Computational Biology)*,   19(Suppl. 2):ii16-ii25, 2003.   [full text] - **SpaMo** Tom Whitington, Martin C. Frith, James Johnson, and Timothy L. Bailey   "Inferring transcription factor complexes from ChIP-seq data",   *Nucl. Acids Res.* 39(15):e98, 2011.   [full text] - **TOMTOM** Shobhit Gupta, JA Stamatoyannopolous,   Timothy Bailey and William Stafford Noble,   "Quantifying similarity between motifs",   *Genome Biology*, 8(2):R24, 2007.   [full text]   Related papers GOMO related   - Mikael Boden and Timothy L. Bailey,   "Associating transcription factor binding site motifs with target Go terms and target genes",   *Nucl. Acids Res*,   36, 4108-4117, 2008.   [full text]   MAST related   - Timothy L. Bailey and Michael Gribskov,   "Score distributions for simultaneous matching to multiple motifs"   *Journal of Computational Biology*,   Vol. 4, pp. 45-59, 1997.   [pdf] - Timothy L. Bailey and Michael Gribskov,   "Methods and statistics for combining motif match scores"   *Journal of Computational Biology*,   Vol. 5, pp. 211-221, 1998.   [pdf]   MEME related   - Timothy L. Bailey and Charles Elkan,   "Fitting a mixture model by expectation maximization to   discover motifs in biopolymers",   *Proceedings of the Second International Conference on Intelligent Systems   for Molecular Biology*,   pp. 28-36, AAAI Press, Menlo Park, California, 1994.   [postscript]   [pdf] - Timothy L. Bailey and Charles Elkan,   "Fitting a mixture model by expectation maximization to   discover motifs in biopolymers",   UCSD Technical Report, CS94-351, March, 1994, University of California at San Diego.   [pdf] - Timothy L. Bailey,   "Discovering motifs in DNA and protein sequences: The   approximate common substring problem",   Ph.D. dissertation, University of California at San Diego, 1995.   [pdf] - Timothy L. Bailey and Charles Elkan,   "Unsupervised Learning of Multiple Motifs in Biopolymers using EM"   *Machine Learning*,   21(1-2):51-80, October, 1995.   [pdf] - Timothy L. Bailey and Charles Elkan,   "The value of prior knowledge in discovering motifs with MEME",   *Proceedings of the Third International Conference on Intelligent Systems for Molecular Biology*,   pp. 21-29, AAAI Press, Menlo Park, California, 1995.   [pdf] - Timothy L. Bailey and Michael Gribskov,   "The megaprior heuristic for discovering protein sequence patterns"",   *Proceedings of the Fourth International Conference on Intelligent Systems for Molecular Biology*,   pp. 15-24 AAAI Press, Menlo Park, California, 1996.   [pdf] - William N. Grundy, Timothy L. Bailey and Charles P. Elkan,   "ParaMEME: A Parallel Implementation and a Web Interface   for a DNA and Protein Motif Discovery Tool",   *Computer Applications in the Biological Sciences (CABIOS)*,   Vol. 12(4), pp. 303-310, 1996.   [pdf] - Timothy L. Bailey, Michael E. Baker and Charles P. Elkan,   "An artificial intelligence approach to motif discovery in protein sequences:   application to steriod dehydrogenases",   *Journal of steroid biochemistry and molecular biology*,   Vol. 62, 1997. - Martin Tompa, Nan Li, Timothy L. Bailey, George M. Church, Bart De Moor,   Eleazar Eskin, Alexander V. Favorov, Martin C. Frith, Yutao Fu, W. James Kent,   Vsevolod J. Makeev, Andrei A. Mironov,, William S. Noble,   Giulio Pavesi, Graziano Pesole, Mireille Regnier, Nicolas Simonis, Saurabh Sinha,   Gert Thijs, Jacques van Helden, Mathias Vandenbogaert, Zhiping Weng, Christopher Workman,   Chun Ye and Zhou Zhu.   "Assessing computational tools for the discovery of   transcription factor binding sites."   *Nature Biotechnology*,   23(1), 137-144, 2005.   [full text] - Timothy L. Bailey, Nadya Williams, Chris Misleh, and Wilfred W. Li,   "MEME: discovering and analyzing DNA and protein sequence motifs"   *Nucleic Acids Research*,   Vol. 34, pp. W369-W373, 2006.   [html]   [pdf]   TOMTOM related   - Emi Tanaka, Timothy L. Bailey, Charles E. Grant, William S. Noble, and Uri Keich,   "Improved similarity scores for comparing motifs"   *Bioinformatics*   27(12): 1603-1609, 2011.   [full text]   Other   - William N. Grundy, Timothy L. Bailey, Charles P. Elkan and Michael E. Baker.   "Hidden Markov Model Analysis of Motifs in Steroid Dehydrogenases and their Homologs"   *Biochemical and Biophysical Research Communications*,   Vol 231, pp. 760-766, 1997.   [pdf] - William N. Grundy, Timothy L. Bailey, Charles P. Elkan and Michael E. Baker.   "Meta-MEME: Motif-based Hidden Markov Models of Protein Families"   *Computer Applications in the Biological Sciences (CABIOS)*,   Vol. 13(4), pp. 397-406, 1997.   [pdf] - Michael E. Baker, William N. Grundy, and Charles P. Elkan,   "Spinach CSP41, an   mRNA-binding protein and ribonuclease, is homologous to   nucleotide-sugar epimerases and hydroxysteroid dehydrogenases",   *Biochemical and Biophysical Research Communications*   248(2), 250-254, 1998.   [pdf] - Michael E. Baker, William N. Grundy, and Charles P. Elkan,   "A common ancestor for a   subunit in the mitochondrial proton-translocating NADH:ubiquinone   oxidoreductase (complex I) and short-chain   dehydrogenases/reductases",   *Cellular and Molecular Life Sciences*,   Vol. 55(3), 450-455, 1999. - John Hawkins, Charles Grant, William S. Noble, and Timothy L. Bailey,   "Assessing   phylogenetic motif models for predicting transcription factor binding sites"   *Bioinformatics   (Proceedings of the Intelligent Systems for Molecular Biology Conference)*,   25(12), i339--347, 2009.   [full text] - Gabriel Cuellar-Partida, Fabian A. Buske, Robert C. McLeay, Tom Whitington,   William Stafford Noble, and Timothy L. Bailey,   "Epigenetic priors for identifying active transcription   factor binding sites",   *Bioinformatics* 28(1): 56-62, 2012   [pdf] |
